# Oral Mucosa and Saliva Alterations Related to Vape

**DOI:** 10.1002/cre2.926

**Published:** 2024-07-05

**Authors:** Bruna Fernandes do Carmo Carvalho, Natália de Carvalho Faria, Letícia Foiani, Gabrielle Luana Jimenez Teodoro Nepomuceno, Desirée Rosa Cavalcanti, Mônica Ghislaine Oliveira Alves, Herculano da Silva Martinho, Mário Pérez‐Sayáns, Janete Dias Almeida

**Affiliations:** ^1^ Universidade Estadual Paulista (UNESP) Instituto de Ciência e Tecnologia e of Science and Technology Câmpus São José dos Campos São Paulo Brazil; ^2^ Centro de Ciências Naturais e Humanas Universidade Federal do ABC Santo André São Paulo Brazil; ^3^ Oral Specialties Center of Suzano Stomatology Service—Suzano Sao Paulo Brazil; ^4^ Technology Research Center (NPT) Universidade Mogi das Cruzes Mogi das Cruzes São Paulo Brazil; ^5^ Oral Medicine, Oral Surgery and Implantology Unit (MedOralRes), Faculty of Medicine and Dentistry Universidade de Santiago de Compostela Santiago de Compostela Spain; ^6^ Instituto de Investigación Sanitaria de Santiago (IDIS), ORALRES Group Santiago de Compostela Spain; ^7^ Instituto de los materiales de Santiago de Compostela (iMATUS) Santiago de Compostela Spain

**Keywords:** electronic nicotine delivery systems, Fourier transform infrared spectroscopy, mouth, nicotine, saliva

## Abstract

**Objectives:**

Electronic nicotine delivery systems (e‐cigarette, pod, and vape) are currently among the tobacco consumption of adolescents and young adults. The aim is to show oral mucosa and saliva alterations related to vape.

**Material and Methods:**

A vape‐user patient, presenting a white plaque in the posterior region of the hard palate, underwent clinical examination, sialometry, pH evaluation, and excisional biopsy of the white lesion. Molecular changes in saliva and vape liquid were analyzed by vibrational spectroscopy.

**Results:**

The histopathological analyses showed hyperparakeratosis without dysplasia. Formaldehyde, ketones, and aromatic hydrocarbon species were identified in e‐cig liquid by the FTIR.

**Conclusions:**

The use of vape may be related to the development of hyperkeratotic lesions in the oral mucosa as well as significantly modify the patient's salivary patterns as the vape liquid presents carcinogenic and cytotoxic components in its composition.

## 1 Introduction

The impact of tobacco use on the oral and systemic health of individuals is already well described in the literature, as well as the risk associated with the development of oral lesions, including oral cancer. However, much has been discussed about the very attractive electronic cigarettes or vapes (e‐cigs), which are currently among the tobacco consumption of adolescents and young adults (Walley et al. 2019; World Health Organization 2023).

The use of these electronic devices has gained popularity among young people, including those who have never smoked cigarettes (Overbeek et al. 2020). In this context, the purpose of this communication is to draw attention to the oral and salivary changes observed in a vape user.

A 26‐year‐old male patient sought care complaining of a rough white plaque on the hard palate that had evolved for 1 month. He mentioned being a former smoker of industrialized cigarettes (about 30 cig/per day, over 8 years). For 4 years, he used vape exclusively and daily, with a nicotine concentration of 50 mg/mL, more than 15 times a day, for about 2 continuous minutes with about 4 mL of fruity or sweetened essence. On average, this corresponds to 200 mg of nicotine consumption per day which corresponds up to five times the nicotine content of a pack of cigarettes.

**Figure 1:**
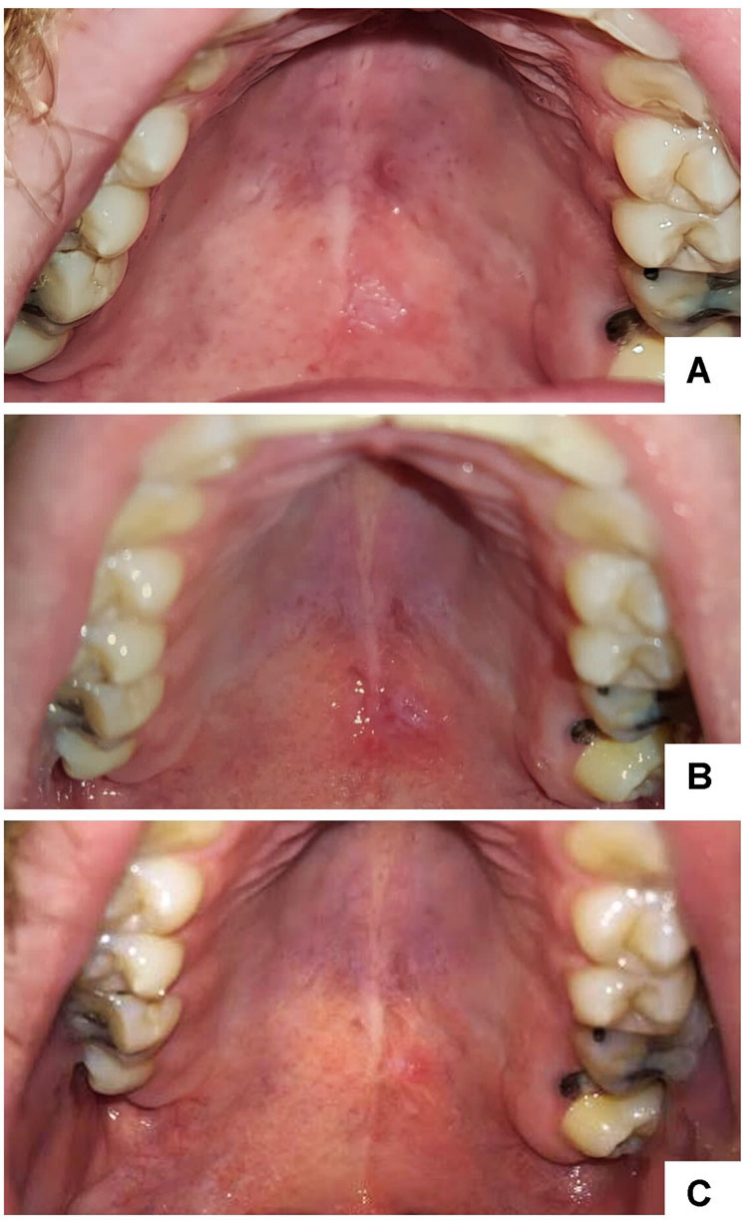
Clinical aspects. (A) Initial clinical findings, (B) 1‐month follow up, and (C) 6 months follow‐up.

On clinical examination, chronic carious lesions were observed on teeth 26 and 36, presence of residual root of 27 and a well‐defined white plaque, with a rough surface, measuring approximately 1 cm in the posterior region of the palate. In the middle third of the palate, there were papules with a reddish central area, compatible with nicotinic stomatitis (Figure 1A).

**Figure 2:**
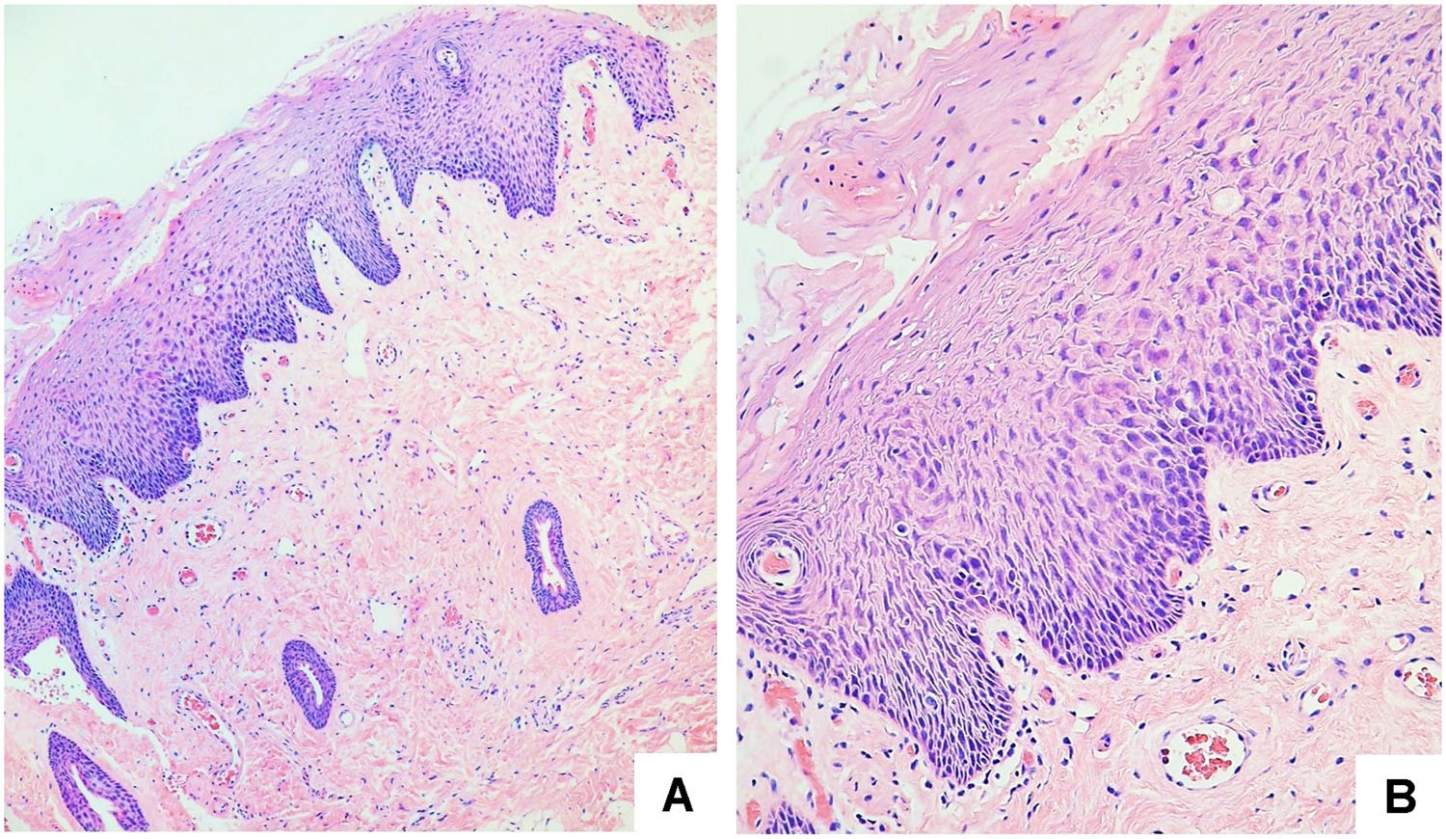
Histopathologic findings (HE staining). Hyperparakeratosis without dysplasia was observed. (A) ×100 magnification and (B) ×400 magnification.

During the anamnesis, our diagnostic work‐up was thoroughly directed to exclude the possibility that the white lesion was related to any other cause. The patient was questioned about the use of inhaled medications, sexual and parafunctional habits, trauma, burns, or the use of any other substances, and did not reveal anything noteworthy that could establish a differential diagnosis. Although the patient did not undergo a period of smoking cessation, he only observed the lesion after he started vaping. Considering the clinical presentation and the patient's history, the diagnostic hypothesis of reactive keratosis versus leukoplakia was established. Thus, an excisional biopsy of the plaque was performed, which presented a histopathological diagnosis of hyperparakeratosis without dysplasia. In addition, the presence of *candida* was not observed (Figure 2).

We additionally collected unstimulated saliva samples and utilized flavored liquid during the assessment. Sialometry readings and pH levels were found to be within normal ranges (0.9 mL/min for sialometry and pH 6.9). Furthermore, the viscosity of saliva was observed to be high, with a thread length of 4 cm.

Fresh liquid from e‐cig was collected directly from the packaging. A similar liquid was also collected from the corresponding reservoir of the e‐cig device in use by the patient. To verify a possible change in the flavoring components, caused by heating the liquid or other process, the flavoring samples were compared with each other.

**Figure 3:**
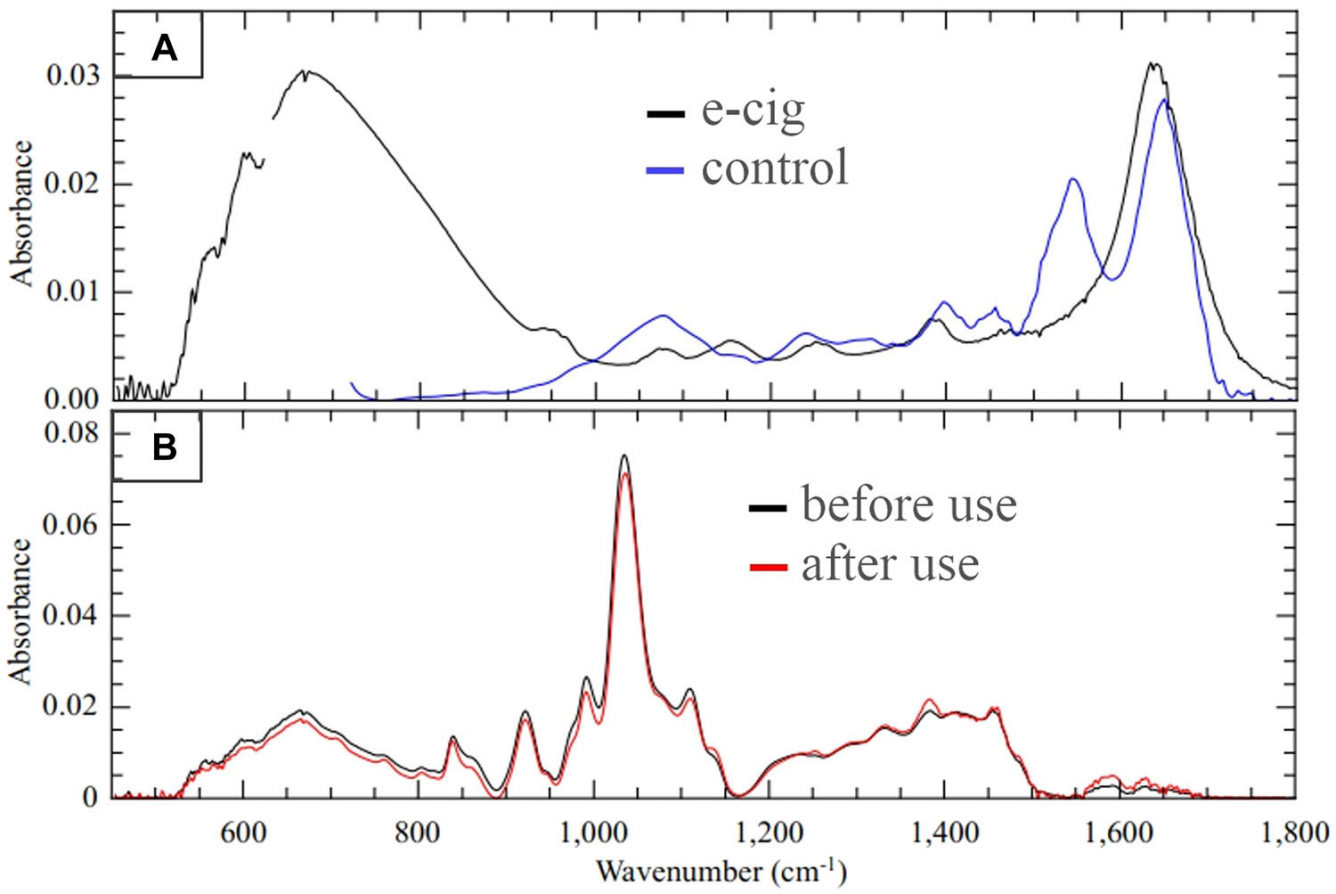
Average spectrum of the samples by FTIR spectroscopy. (A) Vibrational spectroscopy (FTIR) of saliva. Vape user (black line) and control (blue line). (B) Vibrational spectroscopy (FTIR) of e‐cig liquid before (black line) and after use (red line) in vape.

The biochemical composition of the patient's saliva under investigation was compared with standard saliva obtained from a nonsmoker and non‐vape user. One microliter of saliva samples was spread over a platinum substrate and submitted to Fourier‐Transform Infrared Absorption Spectroscopy (FTIR) analysis using micro Attenuated Total Reflectance (ATR) with diamond crystal (60 scans). FTIR spectra presented significant differences between e‐cig and standard saliva in formaldehyde (1750 cm^−1^), ketones (1644–1666 cm^−1^), and aromatic hydrocarbon (3364 cm^−1^) bands (Figure 3A). These compounds are considered by the International Agency for Research on Cancer (IARC) as human carcinogens (International Agency for Research on Cancer [IARC] 2023).

In the visual analysis of the liquids, we observed that there was a change in color, which became brownish after the heating generated by the device. The brownish color is suggestive of carbonization, which is also represented in the cotton and in the resistance of the device. However, when evaluated using FTIR, no structural alterations were observed. The liquid sample, after being heated, maintained the peaks of the initial sample (Figure 3B).

During the 15‐day postoperative follow‐up, a delay in healing was observed. The patient was re‐assessed 1 month after the biopsy and the wound was still in the healing phase (Figure 1B). After 6 months of follow‐up, only the presence of a surgical scar was observed (Figure 1C).

Young individuals are increasingly exposed to vaping at earlier ages. Moreover, frequent usage and high nicotine doses contribute to their growing dependence on these devices (Loukas et al. 2022; Overbeek et al. 2020; Vogel et al. 2019).

E‐cigarette users may experience oral alterations, including xerostomia, hairy tongue, candidiasis, and angular cheilitis, alongside a dysregulated inflammatory response that contributes to the pathogenesis of periodontal diseases (Bardellini et al. 2018; Javed et al. 2017; Ralho, Coelho, and Ribeiro 2019). White lesions, such as leukoplakia, reactive keratosis, and nicotinic stomatitis, similar to those described in the present report, have been documented in e‐cigarette users (Bardellini et al. 2018).

White lesions in the oral cavity can originate from various etiologies, leading to their classification into distinct groups, including developmental changes, metabolic alterations, reactive responses, infectious causes, immune‐mediated conditions, idiopathic origins, potentially malignant lesions, and neoplastic growths. This categorization aids in establishing diagnostic hypotheses (Woo, Grammer, and Lerman 2014).

We deem it essential to include reactional keratosis as a diagnostic hypothesis, given the patient's use of a device that produces substantial steam at elevated temperatures, potentially resulting in mucosal damage.

Leukoplakia, classified as an oral potentially malignant disorder, is primarily diagnosed through exclusionary criteria and is notably linked to tobacco consumption (Warnakulasuriya, Kujan, and Aguirre‐Urizar 2021). Histopathologically, leukoplakia may manifest with or without epithelial dysplasia (Warnakulasuriya, Kujan, and Aguirre‐Urizar 2021). While this lesion was initially considered in the diagnostic hypothesis, current literature offers limited clinical data regarding the association between e‐cigarette use and the development of oral potentially malignant disorders (Schwarzmeier, da Cruz, and Ferreira 2021). Consequently, establishing a direct cause‐and‐effect relationship remains challenging (Gallagher, Vargas, and Santos‐Silva 2024).

Although reviews are not conclusive regarding the use of vape and the direct relationship with the development of oral cancer (Wilson et al. 2022), attention should be paid to lesions that look or have the potential to become malignant. To date, only one case of oral cancer in association with vaping, in a young patient, has been published (Klawinski et al. 2021). However, vape cannot be considered harmless (Ralho, Coelho, and Ribeiro 2019; Thiem, Donkiewicz, and Rejaey 2023).

We posit that the delay in healing experienced by the patient in this case may be attributed, in part, to the heat generated by the continued use of vaping post‐surgery. However, we cannot disregard the possibility that flavoring components may also play a role in delaying healing.

Sundar et al. (2016) reported increased oxidative stress, pro‐inflammatory responses, and pro‐senescence in keratinocytes and oral fibroblasts exposed to e‐cigarette vapor. Moreover, they emphasized that these changes could disrupt the wound repair process. Similarly, Alanazi et al. (2018) demonstrated that exposure to vape vapor adversely affects the activity of gingival fibroblasts, altering their morphology, proliferation rate, and migratory capacity.

Although the present investigation presents the limitation of having the description of only one case, we have pieces of evidence that the use of vape may be related to the development of hyperkeratotic lesions in the oral mucosa and that it significantly modifies the patient's salivary patterns as the vape liquid presents carcinogenic and cytotoxic components in its composition.

## Author Contributions

**Bruna Fernandes do Carmo Carvalho:** conceptualization, data curation, funding acquisition, writing–original draft. **Natália de Carvalho Faria:** methodology, investigation, visualization. **Letícia Foiani:** software, validation, writing–review and editing. Gabrielle Luana **Jimenez Teodoro Nepomuceno:** formal analysis, resources, project administration. **Desirée Rosa Cavalcanti:** supervision, visualization. **Mônica Ghislaine Oliveira Alves:** writing–original draft, project administration, validation. **Herculano da Silva Martinho:** conceptualization, methodology, writing–review and editing. **Mario Pérez‐Sayáns:** investigation, resources, funding acquisition. **Janete Dias Almeida:** data curation, software, funding acquisition, writing–review and editing.

## Conflicts of Interest

The authors declare no conflicts of interest.

## Data Availability

The data that support the findings of this study are available from the corresponding author upon reasonable request.
